# Proceedings of the Hahnemann Club of Toronto

**Published:** 1888-04

**Authors:** J. D. Tyrrell

**Affiliations:** Secretary-Treasurer


### PROCEEDINGS OF THE HAHNEMANN CLUB OF TORONTO.

Regular meeting of the Hahnemann Club opened at 8.20 P. M., the President, Dr. Hall, in the chair. We were honored by the presence of R. Hearn, M. D., as guest. After reading and approval of minutes, the following papers on post-partum hemorrhage were presented. * * * * * *

In order to give a change of mental pabulum, and to give time to select other remedies in post-partum, it was decided to take up "Convulsions" at our next meeting. By this term we include apoplexia, eclampsia, epilepsy, etc., *irrespective of pathology or classification*. On the above subject the following remedies were chosen: Bell., Dr. Hall; Cina, Dr. Adams; Acon., Dr. Eadie; Cham., Dr. Evans; Cicuta vir., Dr. Emory; Nux vom., Dr. Hearn; Stram., Dr. Tyrrell.

We are striving to cover the ground of *emergency cases*, wherein there is no time for the family to wait for their own physician if he be out, or for us to *look up* the remedy; and it is because such emergencies are rare that it behooves us to study, arrange, and *learn* beforehand, for by prompt action many a life may be saved and many a convert made to Homœopathy. Once arrange and *fix in our memory* the *characteristics* of the *best proven* and most *important* remedies, we cannot be dismayed by the *sudden* call and our cause will be vindicated.

J. D. Tyrrell, *Secretary-Treasurer.*

